# Prognostic Value of the Hematometabolic Index for Incident Metabolic Syndrome: Findings From a Large Occupational Cohort of Japanese Men

**DOI:** 10.7759/cureus.98288

**Published:** 2025-12-02

**Authors:** Ichiro Wakabayashi, Tomoyuki Nakano, Kaoru Goto, Takashi Daimon, Kuniaki Ishii

**Affiliations:** 1 Department of Preventive Medicine, Hyogo Medical University, School of Medicine, Nishinomiya, JPN; 2 Department of Anatomy and Cell Biology, Yamagata University School of Medicine, Yamagata, JPN; 3 Department of Biostatistics, Hyogo Medical University, School of Medicine, Nishinomiya, JPN; 4 Department of Pharmacology, Yamagata University School of Medicine, Yamagata, JPN

**Keywords:** cardiovascular disease, follow-up study, hematometabolic index, hemoglobin concentration, leukocyte count, metabolic syndrome

## Abstract

Background: We have recently proposed the hematometabolic index (HMI), which was defined as the product of blood hemoglobin concentration and leukocyte count after modifications. HMI was associated with the risk of cardiovascular disease in a cross-sectional study. This study aimed to investigate the relationship between HMI and the onset of metabolic syndrome.

Methods: A retrospective cohort study was conducted on 7254 adult men (31-75 years old) receiving annual health checkups to investigate the relationship between HMI and incident metabolic syndrome. The subjects were divided into four HMI quartile groups, and the crude and adjusted odds ratios of each quartile vs. the 1st quartile group for metabolic syndrome were estimated using logistic regression analysis.

Results: In the baseline analysis using the data of subjects before follow-up, HMI showed significant positive relationships with the components of metabolic syndrome, including visceral obesity, hypertension, dyslipidemia (high triglycerides and/or low high-density lipoprotein cholesterol), and diabetes. The crude odds ratios for metabolic syndrome after a five-year follow-up of the 2nd, 3rd, and 4th quartile groups of HMI versus the 1st quartile group were 1.51 (1.16-1.96), 2.13 (1.66-2.73), and 3.02 (2.38-3.84), respectively, which were significantly higher than the reference level. The odds ratios remained significant after adjusting for age, histories of habitual smoking, alcohol consumption, and regular exercise, and the above components of metabolic syndrome at baseline.

Conclusion: HMI was associated with incident metabolic syndrome in this retrospective cohort study and is a possible predictor of cardiometabolic risk.

## Introduction

Early correction of cardiovascular risk factors is a critical strategy for reducing the risk of cardiovascular diseases, with atherosclerosis and thrombosis playing major roles. Classical cardiovascular risk factors encompass hypertension, dyslipidemia, and diabetes, closely linked to obesity, which promotes atherosclerosis [1]. Metabolic syndrome represents a cluster of predispositions to atherosclerotic cardiovascular diseases involving the aforementioned risk factors and obesity [2]. In addition to these traditional risk factors, polycythemia is believed to elevate the risk of arterial thrombotic disease due to high blood viscosity, leading to increased shear stress in blood vessels that initiates and expedites atherosclerosis and thrombosis [3,4]. In fact, hematocrit, an index of blood viscosity, was positively associated with metabolic syndrome [5,6]. Moreover, an elevated circulating leukocyte count and increased levels of acute-phase proteins in the blood are indicative of the inflammatory process associated with atherosclerosis and serve as markers for cardiovascular disease [7,8].

Based on the evidence indicating that polycythemia and a high leukocyte count are independently linked to cardiometabolic risk, we recently introduced the hematometabolic index (HMI). The HMI is calculated as the product of blood hemoglobin concentration and leukocyte count after modifications, serving as a novel indicator of cardiometabolic risk [9]. In a cross-sectional study, HMI demonstrated associations with metabolic syndrome and cardiovascular risk factors, such as age, smoking, obesity, hypertension, dyslipidemia, and diabetes [10]. However, the longitudinal relationship between HMI and cardiometabolic risk remains uncertain. Therefore, this follow-up study aims to explore the association between HMI and the development of metabolic syndrome in healthy participants.

## Materials and methods

Study population

**Figure 1:**
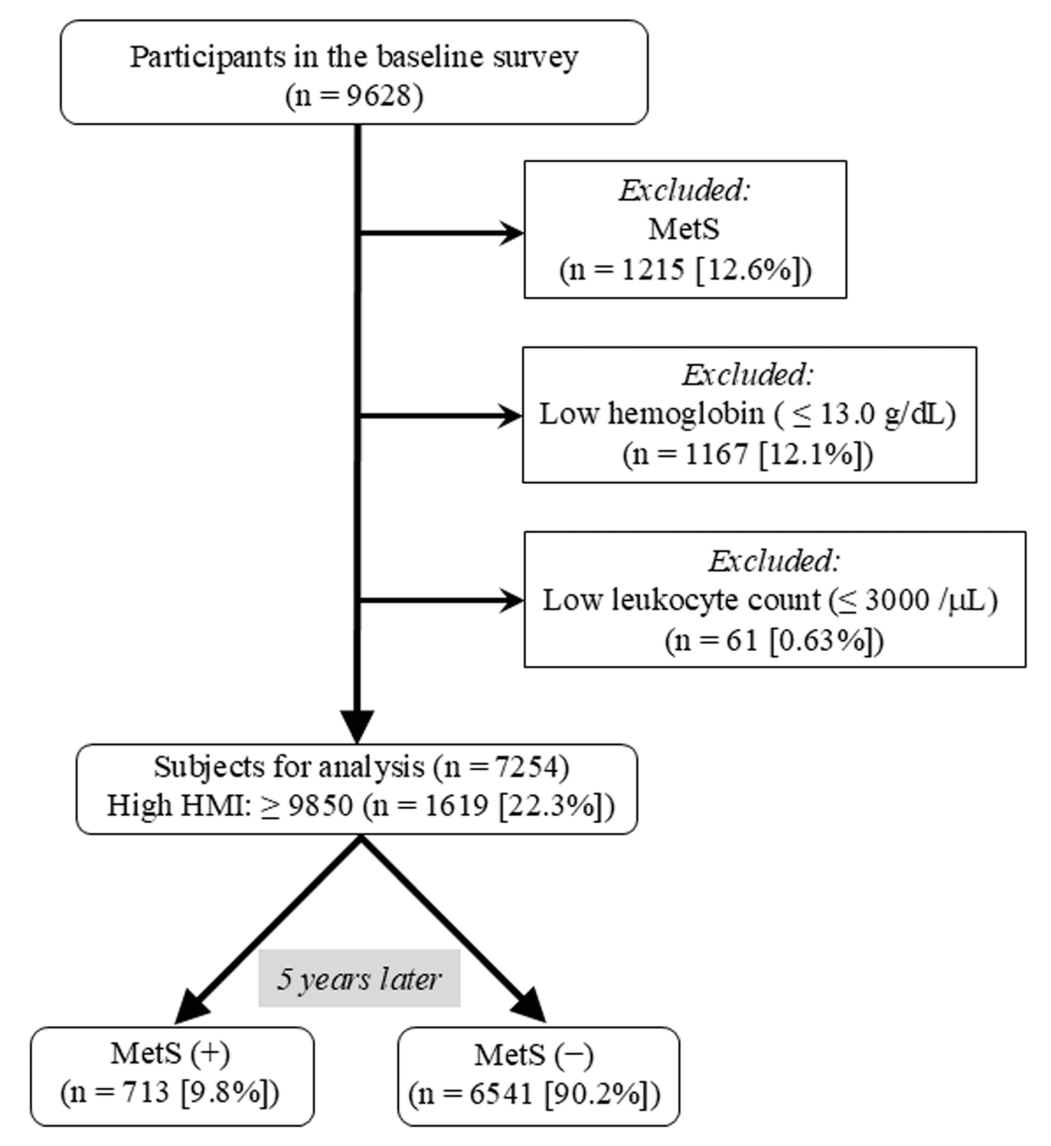
A flowchart illustrating the selection of subjects for analysis from participants at baseline. HMI, hematometabolic index; MetS, metabolic syndrome.

The initial participants comprised 9628 Japanese men (age: median with interquartile range: 50 (44, 56) years) who underwent regular health checkups at workplaces in Yamagata Prefecture, Japan, from January 2018 to December 2018. Five years post the baseline survey, each participant underwent subsequent health assessments at their workplaces. This study was approved by the Hyogo College of Medicine Ethics Committee (No. 3003, dated February 25, 2020). Participants identified with metabolic syndrome based on the criteria described below (n = 1215), anemia (hemoglobin levels of ≤ 13 g/dL (n = 1167)), and/or showing leukocytopenia (leukocyte count of ≤ 3000/microL (n = 61)) during the baseline survey were excluded from the study. Consequently, 7254 participants were included in the analysis. Following the five-year follow-up period, 713 subjects (9.8%) were diagnosed with metabolic syndrome (Figure 1).

Information on lifestyles

Histories of cigarette smoking, alcohol consumption, regular exercise, illness, and therapy for illness were surveyed using questionnaires as described previously [9]. Regarding smokers, in the self-written questionnaire paper, participants were first asked, “Are you a habitual cigarette smoker?” Cigarette smokers were defined as participants who had smoked for six months or longer and had smoked for the past month or longer. Then, the participants who were smokers were further asked, “What is your average cigarette consumption per day?” The response categories for this question were “20 or fewer cigarettes per day”, “21 or more and 40 or less cigarettes per day”, and “41 or more cigarettes per day”. Because the percentage of participants with very heavy cigarette consumption (41 or more cigarettes per day) was very low (0.59% (n = 43)), they were included in the heavy smoking group. The participants were then divided into three groups: nonsmokers, light smokers (20 or fewer cigarettes per day), and heavy smokers (21 or more cigarettes per day). The average alcohol consumption of each participant per week was also reported in the questionnaires. Frequency of habitual alcohol drinking was asked in the questionnaire as “How frequently do you drink alcohol?” Frequency of weekly alcohol drinking was categorized as “every day” (regular drinkers), “sometimes” (occasional drinkers), and “never” (nondrinkers). Participants with a habit of regular exercise were defined as those exercising almost every day for 30 minutes or longer per day.

Measurements of cardiovascular risk factors

Height and body weight were assessed while wearing light clothing during the health examination, and body mass index (BMI) was calculated as weight in kilograms divided by the square of height in meters. Waist circumference was determined at the level of the navel following the guidelines of the Japanese Committee for the Diagnostic Criteria of Metabolic Syndrome [11], and the waist-to-height ratio was calculated by dividing waist circumference in centimeters by height in centimeters. Blood pressure was recorded at rest in a seated position using an oscillometric device (TM-2580; A&D Company, Limited, Tokyo, Japan).

Fasted blood samples were collected from each participant in the morning. Concentrations of the variables in the blood were measured, and HMI was calculated as described previously [9]: hemoglobin concentrations were assessed using the sodium lauryl sulfate hemoglobin method, while leukocyte counts were measured via flow cytometry. The HMI was defined as the product of hemoglobin concentration (g/dL) minus 13.0 and leukocyte count (/microL) minus 3000, and the cut-off value of the HMI was 9850 (AUC: 0.707 (0.663 ~ 0.751)) [9]. Serum triglyceride and HDL cholesterol levels were quantified enzymatically using commercial kits, namely, Pureauto S TG-N and Cholestest N-HDL (Sekisui Medical Co., Ltd, Tokyo, Japan), respectively. Hemoglobin A_1c_ concentration was measured through the latex cohesion method employing a commercial kit (Determiner HbA_1c_; Kyowa Medex, Tokyo, Japan) and calibrated using a formula proposed by the Japan Diabetes Society [12]. The coefficients of variation for reproducibility of each measurement were ≤ 3% for hemoglobin, leukocyte count, and triglycerides, and were ≤ 5% for HDL cholesterol, LDL cholesterol, and hemoglobin A_1c_.

Metabolic syndrome was defined based on the International Diabetes Federation criteria [2] with a slight modification as described previously [9]: the presence of two or more risk factors along with visceral obesity identified by a high waist-to-height ratio. The risk factors considered were visceral obesity (high waist-to-height ratio), hypertension, dyslipidemia (low HDL cholesterol and/or high triglycerides), and diabetes, evaluated by hemoglobin A_1c_ levels. The criteria for each risk factor in metabolic syndrome were as follows: visceral obesity, waist-to-height ratio ≥ 0.5 [13]; hypertension, systolic blood pressure ≥ 140 mmHg and/or diastolic blood pressure ≥ 90 mmHg [14]; low HDL cholesterol, HDL cholesterol < 40 mg/dL; high triglycerides, triglycerides ≥ 150 mg/dL [15,16]; diabetes, hemoglobin A_1c_ ≥ 6.5% [17]. Individuals under drug therapy for hypertension and diabetes were also considered in the definitions of hypertension and diabetes, respectively.

Statistical analysis

Continuous variables were summarized as means with standard deviations or medians with interquartile ranges, as appropriate. Categorical variables were summarized as frequencies and percentages. Participants were sorted by HMI levels in ascending order and then divided into four nearly equal-sized quartiles. Continuous variables were compared among the quartile groups using analysis of variance (ANOVA), followed by Scheffé’s F-test as a post-hoc test. Age and triglyceride levels, which did not exhibit a normal distribution, were compared nonparametrically among the quartile groups of HMI using the Kruskal-Wallis test followed by Steel’s test as a post-hoc test. Categorical variables were compared using Pearson’s chi-square test. The impact of baseline HMI on incident metabolic syndrome was analyzed using a logistic regression model, estimating crude and adjusted odds ratios, with their corresponding 95% confidence intervals. In the multivariable analyses, adjustments were made for age, histories of smoking, alcohol consumption and regular exercise, and a history of therapy for dyslipidemia. Additionally, visceral obesity (high waist-to-height ratio), hypertension, dyslipidemia, and diabetes at baseline were adjusted as covariates as appropriate. All probability (p) values were two-sided, with statistical significance defined as p < 0.05. Statistical analyses were performed using IBM SPSS Statistics for Windows, Version 25.0 (IBM Corp., Armonk, NY, USA).

## Results

Characteristics of the subjects

The characteristics of the participants included in the analysis are presented in Table 1. The median age was 50 years, and the percentages of smokers, drinkers, and subjects exercising regularly were 39.9%, 70.9% and 15.3%, respectively. The percentages of participants with visceral obesity, hypertension, dyslipidemia (hypertriglyceridemia and/or low HDL cholesterolemia), and diabetes were 37.6%, 35.9%, 18.6% and 3.3%, respectively.

**Table 1:** Characteristics of overall subjects and subjects of each quartile group of HMI at the baseline of the study and comparison of each variable in the four HMI quartiles. Presented are numbers, percentages, means with standard deviations, and medians with interquartile ranges. Symbols indicate significant differences from the 1st quartile of HMI (*, p = 0.028; **, p < 0.01). HMI, hematometabolic index.

Variable	1st quartile	2nd quartile	3rd quartile	4th quartile	Overall
Number	1810	1820	1816	1808	7254
Age (years)	52 (46, 57)	51 (45, 57)**	50 (44, 56)**	49 (43, 55)**	50 (44, 56)
Smokers: light, heavy (%)	18.7, 1.5	29.2, 3.9**	37.4, 6.4**	48.5, 14.0**	33.4, 6.5
Alcohol drinkers: occasional, regular (%)	32.2, 31.2	32.9, 41.0**	32.8, 41.8**	31.0, 40.6**	32.2, 38.7
Regular exercise (%)	14.2	16.4	16.9*	13.9	15.3
Height (cm)	164.2 ± 8.8	168.8 ± 7.7**	169.8 ± 6.9**	170.3 ± 6.4**	168.3 ± 7.9
Weight (kg)	60.3 ± 10.8	65.3 ± 10.8**	68.3 ± 10.6**	71.2 ± 12.0**	66.2 ± 11.8
Waist circumference (cm)	79.1 ± 9.0	81.3 ± 8.7**	83.6 ± 8.6**	85.7 ± 9.5**	82.4 ± 9.3
Waist-to-height ratio	0.483 ± 0.057	0.482 ± 0.052	0.493 ± 0.051**	0.503 ± 0.056**	0.490 ± 0.055
High waist-to-height ratio (%)	32.5	32.6	39.7**	45.7**	37.6
Erythrocyte (/microL)	456.0 ± 29.4	480.5 ± 29.8**	496.1 ± 30.5**	516.1 ± 33.5**	487.1 ± 37.9
Hemoglobin (g/dL)	13.93 ± 0.64	14.75 ± 0.64**	15.27 ± 0.66**	15.95 ± 0.77**	14.98 ± 1.00
Leukocyte (/microL)	4961 ± 1153	5611 ± 1052**	6413 ± 1118**	7973 ± 1598**	6238 ± 1680
HMI	1438 ± 777	4053 ± 767**	7151 ± 1077**	14234 ± 5128**	6714 ± 5478
High HMI (%)	0	0	0	89.5**	22.3
Systolic blood pressure (mmHg)	125.3 ± 18.1	127.6 ± 17.7**	129.7 ± 16.7**	132.0 ± 17.9**	128.6 ± 17.8
Diastolic blood pressure (mmHg)	77.4 ± 11.7	79.7 ± 11.8**	81.5 ± 11.5**	83.7 ± 12.0**	80.6 ± 12.0
Hypertension (%)	30.9	33.5	37.8**	41.5**	35.9
Therapy for hypertension (%)	13.3	14.9	15.1	14.9	14.5
Triglycerides (mg/dL)	72 (53, 102)	86 (62, 122)**	97 (71, 134)**	113 (80, 157)**	91 (64, 131)
High triglycerides (%)	9.3	14.8**	18.7**	27.8**	17.6
HDL cholesterol (mg/dL)	70.0 ± 16.3	64.5 ± 15.8**	61.4 ± 14.6**	57.7 ± 14.4**	63.4 ± 16.0
Low HDL cholesterol (%)	1.2	2.9**	3.5**	5.8**	3.3
Dyslipidemia (%)	9.6	15.7**	19.8**	29.1**	18.6
Therapy for dyslipidemia (%)	6.2	6.1	7.2	6.7	6.5
Hemoglobin A_1c_ (%)	5.36 ± 0.42	5.38 ± 0.39	5.43 ± 0.52**	5.51 ± 0.65**	5.42 ± 0.51
Diabetes (%)	2.3	2.1	3.2	5.4**	3.3
Therapy for diabetes (%)	1.8	1.9	2.0	3.4**	2.3

Comparison of each variable in the four quartile groups of HMI

Table 1 presents the results of comparing each variable related to cardiovascular risk factors across the four HMI quartiles. Age was slightly but significantly lower in the 2nd, 3rd, and 4th quartile groups compared to the 1st quartile group, showing a trend of decreasing age with higher quartiles. The proportions of light and heavy smokers were significantly higher in the 2nd, 3rd, and 4th quartile groups than in the 1st quartile group, with a tendency to increase across quartiles. The percentage of regular drinkers was significantly higher in the 2nd, 3rd, and 4th quartile groups compared to the 1st quartile group. Height, body weight, waist circumference, and the proportions of individuals with a large waist circumference were significantly higher in the 2nd, 3rd, and 4th quartile groups than in the 1st quartile group, showing an increasing trend with higher quartiles. The waist-to-height ratio was significantly higher in the 3rd and 4th HMI quartile groups than in the 1st quartile group. Systolic and diastolic blood pressure levels, triglyceride levels, and the proportions of individuals with high triglycerides, low HDL cholesterol, and dyslipidemia were significantly higher in the 2nd, 3rd, and 4th quartile groups compared to the 1st quartile group. These parameters also showed a tendency to increase across quartiles. HDL cholesterol was significantly lower in the 2nd, 3rd, and 4th quartile groups than in the 1st quartile group, showing a decreasing trend with higher quartiles. The proportions of hypertension and hemoglobin A_1c_ levels were significantly higher in the 3rd and 4th quartile groups compared to the 1st quartile group, with an increasing trend across quartiles. The proportions of participants with diabetes and those undergoing diabetes therapy were significantly higher in the 4th quartile group compared to the 1st quartile group.

Incidence of metabolic syndrome across HMI quartiles

**Figure 2:**
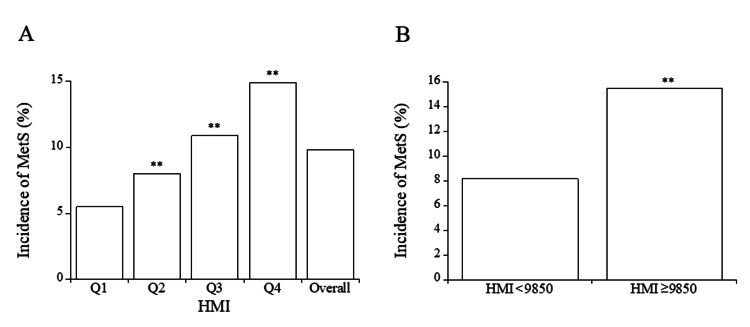
(A) Comparison of incidence of metabolic syndrome in the four quartile groups of HMI. (B) Comparison of incidence of metabolic syndrome in the groups with and without high HMI (≥ 9850). (A) Symbols indicate significant differences from the 1st quartile group of HMI (**, p < 0.01). (B) Symbols indicate a significant difference from the subjects without high HMI (**, p < 0.01). HMI, hematometabolic index; MetS, metabolic syndrome; Q1, 1st quartile; Q2, 2nd quartile; Q3, 3rd quartile; Q4, 4th quartile.

After a five-year follow-up period, 9.8% of all participants developed metabolic syndrome. The incidence of metabolic syndrome was notably higher in the 2nd, 3rd, and 4th quartile groups of HMI compared to the 1st quartile group, showing a trend of increasing incidence with higher quartiles (Figure 2A). Using the cutoff value of 9850 for HMI, 22.3% of the subjects exhibited high HMI levels at baseline (Table 1). Furthermore, individuals with high HMI had a significantly greater risk of developing metabolic syndrome compared to those without high HMI (Figure 2B).

Odds ratios for metabolic syndrome across HMI quartiles

The results of the logistic regression analysis of the relationship between HMI and metabolic syndrome are presented in Table 2. Crude odds ratios for metabolic syndrome of the 2nd, 3rd, and 4th quartile groups of HMI compared to the 1st quartile were significantly higher than the reference level and increased with each quartile (1.51 (2nd quartile) vs. 2.13 (3rd quartile) vs. 3.02 (4th quartile)). After adjusting for age, histories of smoking, alcohol consumption, and regular exercise, and a history of therapy for dyslipidemia ("Multivariable-1" in Table 2), the odds ratios for metabolic syndrome were similar to the corresponding crude odds ratios. Upon further adjustment for the components of metabolic syndrome (obesity, hypertension, dyslipidemia, and diabetes) in "Multivariable-2" in Table 2, the aforementioned odds ratios remained statistically significant, although the odds ratio for each quartile was lower than the corresponding crude odds ratio.

**Table 2:** Longitudinal relationships between HMI and metabolic syndrome in logistic regression analysis. The odds ratios for metabolic syndrome in each quartile compared to the 1st quartile of HMI and the odds ratios for metabolic syndrome in subjects with high HMI compared to subjects without high HMI are presented. In the multivariable analysis, two different adjustments were performed. In one adjustment (Multivariable-1), age, current smoking status, alcohol consumption, regular exercise, and a history of therapy for dyslipidemia were used as explanatory variables. In the other adjustment (Multivariable-2), visceral obesity (high waist-to-height ratio), hypertension, dyslipidemia, and diabetes at the baseline of the study, in addition to age, current smoking status, alcohol consumption, regular exercise, and a history of therapy for dyslipidemia, were used as explanatory variables. Subjects with high HMI were defined as those with an HMI of 9850 or higher [9]. Asterisks indicate significant differences from the reference level of 1.00 (*, p = 0.024; **, p < 0.01). HMI, hematometabolic index.

Condition	OR (95% confidence interval)
2nd vs. 1st quartiles of HMI	
Univariable	1.51 (1.16 ~ 1.96)**
Multivariable-1	1.58 (1.20 ~ 2.06)**
Multivariable-2	1.38 (1.04 ~ 1.83)*
3rd vs. 1st quartiles of HMI	
Univariable	2.13 (1.66 ~ 2.73)**
Multivariable-1	2.15 (1.66 ~ 2.79)**
Multivariable-2	1.52 (1.15 ~ 2.00)**
4th vs. 1st quartiles of HMI	
Univariable	3.02 (2.38 ~ 3.84)**
Multivariable-1	3.09 (2.38 ~ 4.02)**
Multivariable-2	1.72 (1.30 ~ 2.28)**
Subjects with vs. subjects without high HMI	
Univariable	2.07 (1.76 ~ 2.44)**
Multivariable-1	2.10 (1.77 ~ 2.51)**
Multivariable-2	1.35 (1.12 ~ 1.63)**

When the subjects were divided into two groups, with and without high HMI, using a cutoff value of 9850, the odds ratio of the group with versus without high HMI was significantly higher than the reference level in both univariable and multivariable analyses (Table 2).

## Discussion

Approximately 10% of the subjects developed metabolic syndrome after a five-year follow-up, with the incidence and odds ratios for metabolic syndrome increasing with higher quartiles of HMI. This study is the first to establish an association between HMI and the development of metabolic syndrome. HMI correlates with age, smoking habits, and alcohol consumption [10]. In a multivariable analysis adjusted for age and lifestyle factors, such as smoking, alcohol consumption, and regular exercise, the odds ratio for each quartile compared to the 1st quartile was consistent with the crude analysis. Thus, the relationship between HMI and metabolic syndrome does not seem to be confounded by age or lifestyle factors. In a multivariable analysis considering metabolic syndrome components such as obesity, hypertension, dyslipidemia, and diabetes, the odds ratios for metabolic syndrome in the 2nd, 3rd, and 4th quartile groups of HMI compared to the 1st quartile group were lower than the crude odds ratios but remained statistically significant (refer to Table 2). These risk factors likely confound the association between HMI and metabolic syndrome as they are both components of metabolic syndrome [2] and are associated with HMI [10]. Therefore, in this follow-up study, HMI was found to be associated with an increased risk of metabolic syndrome in men in a dose-dependent manner, and this association was shown to be, at least in part, independent of the components of metabolic syndrome at the baseline of this study. When a cutoff value of 9850 [9] was used for high HMI, about 22% of the subjects showed high HMI, and the crude odds ratio for metabolic syndrome was about 2.00 in subjects with high HMI versus those without high HMI. Further studies of comparison of HMI with other predictors and established risk scores, including lipid-related indices and insulin resistance-related markers, are needed to confirm the usefulness of HMI as a predictor of cardiovascular disease.

This study had some limitations. Individuals with low hemoglobin levels and/or low leukocyte counts in the blood were excluded from the analysis. Thus, HMI is not used in patients with anemia or leukopenia, although these conditions are generally not thought to be associated with the progression of atherosclerosis. The subjects of this study were all men because the proposed HMI is used only for men [9]. Further studies are needed to develop a similar index reflecting both high hemoglobin concentration and leukocyte count for predicting cardiometabolic risk in women, although hemoglobin concentrations are affected by menstrual status, and anemia is significantly more frequent in women than in men. The subjects of this study were Japanese, and thus, HMI needs to be investigated in other ethnic groups. Because we used the database of annual health checkups for workers, there is a possibility of biases caused by the healthy worker effect. In the multivariable analyses, age, lifestyle histories, and components of metabolic syndrome were used as confounders for adjustment. However, there are other possible confounders of the relationship between HMI and metabolic syndrome, such as diet, nutrition, and socioeconomic factors, including education, occupation, and income [18]. The habit of alcohol drinking, which has been reported to be associated with HMI [10], was evaluated by the frequency of individual weekly drinking, but not by the amount of alcohol intake. Serum level of gamma-glutamyl transferase (GGT) is used as a biomarker for sustained excessive alcohol intake [19]. The medians with interquartile intervals of serum GGT levels (U/L) were 22 (16, 35) in non-drinkers, 30 (20, 50) in occasional drinkers, and 46 (29, 81) in regular drinkers in the present study. Thus, the GGT levels tended to increase with an increase in the frequency of alcohol consumption, indicating that the classification of alcohol consumption also reflects the amount of alcohol intake. In addition, there are other limitations of this study, such as the single follow-up time point without capturing the time of metabolic syndrome onset and the absence of clinical endpoints using cardiovascular events.

## Conclusions

The HMI showed a dose-dependent association with the incidence of metabolic syndrome in men in the general population. This association was independent of age and lifestyle factors and remained significant even after adjustment for the components of metabolic syndrome at the baseline of the study. Therefore, HMI may be a predictor of cardiometabolic risk.
